# Transient cardiac arrest in a child with Down syndrome during anesthesia induction with sevoflurane: a case report

**DOI:** 10.1186/s40981-016-0043-8

**Published:** 2016-08-08

**Authors:** Kentaro Nogami, Shogo Taniguchi, Kyoko Togami

**Affiliations:** Section of Anesthesiology, Department of Diagnostics and General Care, Fukuoka Dental College, 2-15-1, Tamura, Sawara-Ku, Fukuoka-shi, 814-0193 Fukuoka Japan

**Keywords:** Down syndrome, Sevoflurane, General anesthesia, Cardiac arrest, Pediatric patient

## Abstract

**Background:**

This report describes a case of transient cardiac arrest in a child with Down syndrome. The cardiac arrest occurred during induction of anesthesia with sevoflurane. To the best of our knowledge, this is the first such report.

**Case presentation:**

A 14-year-old boy was scheduled to undergo dental treatment under general anesthesia because of his mental disorder. He had congenital atrial and ventricular septal defects and patent ductus arteriosus, which had been repaired previously. Therefore, we anticipated no problems with his cardiovascular system during the perioperative period. Because the sedation administered before the insertion of an intravenous catheter and arterial line was insufficient to induce an anesthetic effect, general anesthesia was induced by using a mixture of sevoflurane (5 %) with oxygen in nitrous oxide. A few minutes after the induction of anesthesia, the patient unexpectedly experienced bradycardia (heart rate <30 beats/min), and his electrocardiography findings indicated asystole. After a few minutes of cardiopulmonary resuscitation, the patient’s heart rate returned to normal.

**Conclusions:**

We postulated that the asystole was triggered by a dysfunction in the autonomic cardiac regulation and sympathetic activation, which often occurs in patients with Down syndrome, and due to the use of high concentrations of sevoflurane. In future cases of pediatric patients with Down syndrome, with or without heart disease, the concentration of sevoflurane administered during surgery should be increased gradually. Moreover, an intravenous catheter should be promptly inserted to administer anticholinergic drugs as quickly as possible in order to prevent transient cardiac arrest.

## Background

Down syndrome is one of the most common genetic disorders associated with trisomy in chromosome 21, with an incidence of about 1 in 800 live births. Approximately 50 % of children with Down syndrome are born with congenital heart diseases [1, 2]. Consequently, the perioperative management of patients with both Down syndrome and heart disease is focused on the management of the risk factors of cardiovascular disease. General anesthesia with sevoflurane is routinely used in children with Down syndrome and congenital heart diseases because of its favorable hemodynamic stability. However, some cases of severe bradycardia and hypotension during sevoflurane induction have been reported in children with Down syndrome, even in those without congenital heart disease [3–6]. Such complications appear to be related to the dysfunction of the sympathetic nervous system, which is peculiar among patients with Down syndrome [3–6]. Although there have been previous reports of bradycardia and hypotension associated with sevoflurane use in children with Down syndrome, there are no reports on transient cardiac arrest occurring without a preceding cardiac event, hypoxia, or hypercapnia. We present the case of a pediatric patient with Down syndrome who experienced transient cardiac arrest during anesthesia induction with sevoflurane.

## Case presentation

The Institutional Review Board of Fukuoka Dental College approved this study.

The patient was a 14-year-old boy (height = 150 cm, weight = 54 kg) with Down syndrome, atrial septal defect (ASD), ventricular septal defect (VSD), and patent ductus arteriosus (PDA). His congenital heart defects were surgically repaired at 5 months of age. Thereafter, he was followed up once a month with echocardiography. No structural or mechanical problems and dysfunction were noted in his cardiovascular system, such as a left-right shunt. The most recent findings of his echocardiogram were as follows: all chambers were balanced; good wall motion; no asynergy; normal interventricular septum; left ventricular ejection fraction, 85 %; left ventricular end-diastolic dimension, 42 mm; no valve regurgitations; and no abnormal flow resulting from the ASD, VSD, and PDA. The attending physician of the pediatric cardiology section categorized the patient’s condition as grade 1 heart failure according to the New York Heart Association functional classification system. Therefore, we assessed the patient in accordance with the diagnosis of the attending physician. The patient’s intelligence quotient was 35, and his intellectual level was that of a 1.5-year-old child.

**Fig. 1:**
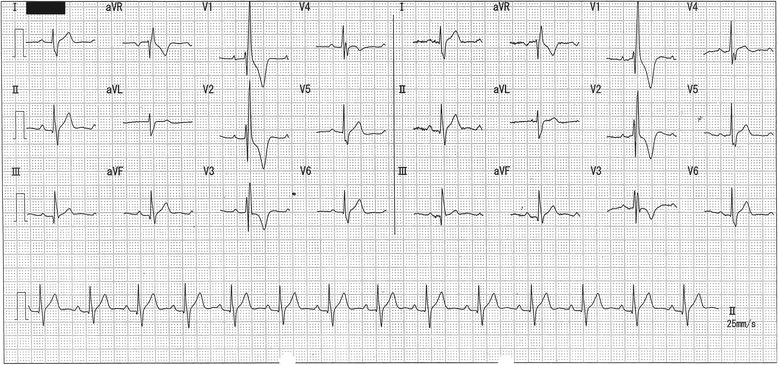
Electrocardiogram

The patient had many dental caries, as observed during examination at the Fukuoka Dental College Hospital. However, because he was uncooperative owing to his condition and our attempts at physical restraint had failed (he violently refused our intervention), he was scheduled for dental treatment under general anesthesia. At our hospital, we recommend that all patients undergo routine checkup, including blood tests, elective cardiograms, chest radiography, and urine tests. However, because of the patient’s violent behavior, we were unable to perform any of the aforementioned tests. Therefore, we referred to the data obtained by the attending physician during the routine checkup of his cardiac condition. According to the preanesthetic examination that was performed at the other institution, the patient had no abnormalities in his blood or urine. A complete right bundle branch block (RBBB) was the only abnormality observed on the electrocardiogram (ECG) (Fig. 1). The patient was not regularly taking prescription medication for his congenital heart disease. His blood pressure (BP), heart rate (HR), peripheral oxygen saturation (SpO_2_), and body temperature could not be measured because of his violent behavior.

The patient’s parents were informed about the risks of general anesthesia before they provided consent. We determined that the perioperative risk in this patient was similar to that in other patients who had undergone corrective surgery for ASD, VSD, and PDA because there were no structural or mechanical problems with his cardiovascular system. He did not present with any risk factors for complications resulting from intubation, such as macroglossia, micronesia, and brevicollis. Although we did not check for dislocation of the first cervical vertebra, it did not seem necessary to restrain his head for airway management. He consumed no food for 6 h and no liquid for 2 h before the operation. We decided to intramuscularly inject midazolam before surgery and to gradually and incrementally induce anesthesia by means of inhalation of sevoflurane with oxygen and nitrous oxide through a face mask, after which an intravenous catheter and arterial line were inserted. To prevent infective endocarditis, we also planned to administer intravenous antibiotics during the operation.

At 9:30 a.m. on the day of the operation, the patient was administered 10 mg midazolam intramuscularly as a sedative. At 10:00 a.m., he was brought to the operating room on a gurney. However, he was not sedated because the midazolam had not taken effect. Moreover, because of his inability to emotionally cope with the environmental change, he acted violently against physical restraint and was in danger of falling from the operating table; therefore, it became necessary to perform anesthesia induction immediately. We therefore administered sevoflurane (5 %) to induce general anesthesia as rapidly as possible. We intended to decrease the concentration of sevoflurane as soon as the patient’s movements were controlled. Because of the patient’s violent behavior, we could not measure his BP, HR, SpO_2_, and body temperature and could not perform the cardiac evaluation with insertion of an intravenous catheter and arterial line before the induction. The anesthetist who performed the anesthesia induction had 15 years of experience at the time. Three anesthetists, who are all co-authors of this paper, performed the anesthesia induction.

**Fig. 2:**
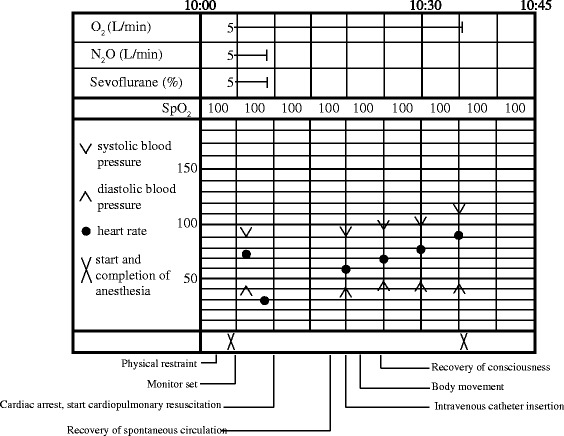
Anesthetic chart

**Fig. 3:**
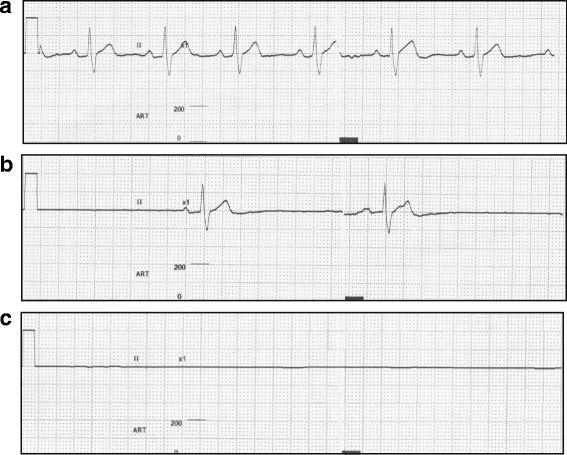
Electrocardiographic changes before CPR. **a** Initial electrocardiogram: HR=75 bpm. **b** Approximately one minute and few seconds after induction: HR=20 bpm. **c** Just before cardiopulmonary resuscitation: HR=0 bpm

**Fig. 4:**
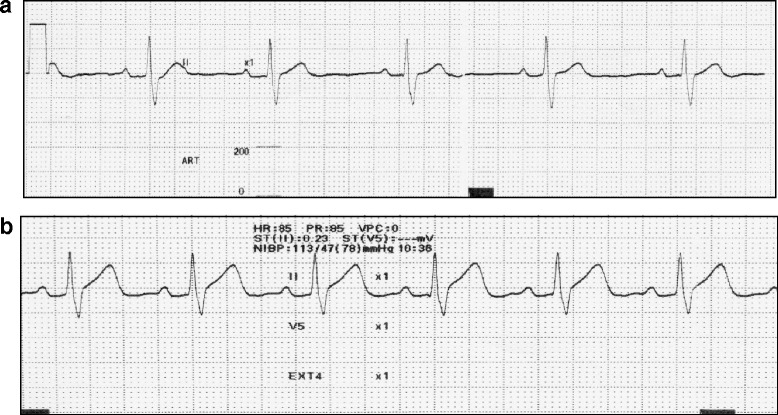
Electrocardiographic changes after CPR. **a** After cardiopulmonary resuscitation: HR=50 bpm. **b** Before leaving the operation room: HR=95 bpm

Approximately 1 min after the induction, the patient’s consciousness and combative behavior gradually decreased. His respiration ratio did not change, and his tidal volume progressively subsided. We then began performing assisted ventilation, which was achieved by using a face mask (Fig. 2). The initial vital signs were as follows: BP = 90/42 mm Hg, HR = 75 beats per minute (bpm), and SpO_2_ = 100 %. The ECG initially showed a normal sinus rhythm (Fig. 3). However, a few seconds later, his ECG indicated severe bradycardia (HR of <30 bpm) and gradually showed prolonged R-R intervals, immediately followed by asystole (Fig. 3). Although we could manage his airway and ventilation, we did not detect a pulse in the carotid artery. We immediately ceased the administration of sevoflurane and nitrous oxide and began cardiopulmonary resuscitation (CPR) with a combination of 15 chest compressions and two ventilations (Fig. 2). We thought that it was unnecessary to perform tracheal intubation during the initial ventilation because controlled ventilation was achievable through a face mask during CPR. Therefore, we performed tracheal intubation during the switch between rescuers in order to minimize interruptions in the chest compressions. A few minutes after CPR, the previous chest compression waveform on the ECG changed, as the patient’s heartbeat and breathing returned (Fig. 4). We therefore stopped CPR, inserted an intravenous catheter in the antecubital vein, and continued ventilation and monitoring until the patient regained consciousness. Throughout this incident, his SpO_2_ did not decrease (Fig. 2). After conferring with the operating dentist, we decided to stop the operation under general anesthesia. After the return of his spontaneous circulation, we decided to perform tracheal intubation while the patient was still unconscious and not spontaneously breathing. The patient regained consciousness relatively early because he only inhaled sevoflurane for a short period. Thereafter, he was transported to the recovery room of the ward (Fig. 4). He was monitored for 1 day at our hospital and was then discharged the next morning without any sequelae. Even after consulting with pediatricians and cardiologists at our hospital, the cause of his cardiac arrest remains unclear.

After this incident, the operating dentist scheduled his dental treatment under sedation using midazolam and physical restraint.

## Discussion

Our case demonstrates the possibility for patients with Down syndrome to experience cardiac arrest during induction of general anesthesia. We believe that the asystole was triggered by the dysfunction in autonomic cardiac regulation and sympathetic activation; these issues are often observed in patients with Down syndrome and after the use of sevoflurane. Other possible causes (including a cardiac event; hypoxia; repaired ASD, VSD, and PDA; and RBBB) were not suspected to be responsible for the cardiac arrest.

The findings of a previous report on anesthesia-related cardiac arrest in children with congenital heart disease showed that the conditions of most of the affected patients were unrepaired (59 %) or palliated (26 %) [7]. Our patient had no structural or mechanical problems with his heart; however, he possibly had other dysfunctions in the cardiovascular system, as has been reported in other patients [8–10]. Agiovlasitis et al. [8] suggested that the decreased HR caused by orthostatic stress in patients with Down syndrome without cardiac disease was related to dysfunction in autonomic cardiac regulation and the blunting of sympathetic activation. Chromosome 21, three copies of which are inherited in Down syndrome, controls the size of certain cells and their number per unit area, which may explain the cardiac dysfunction and blunting of sympathetic activation [9, 10]. Therefore, dysfunctional sympathetic nerve activities may have caused the bradycardia that preceded asystole in our patient.

Bradycardia is the most frequently observed arrhythmia that precedes cardiac arrest in children, according to a registry report on pediatric perioperative cardiac arrest, in which the high incidence of cardiovascular arrest during anesthesia was highlighted [8]. Green et al. [11] found that the onset of bradycardia occurred significantly earlier with the induction of high-concentration sevoflurane than with incremental induction. We suspect that a severe vasovagal reflex caused the asystole subsequent to bradycardia in our patient. Usually, the ECG R-R intervals in the vasovagal reflex prolong gradually and lead to sinus arrest [12]. In our patient, asystole occurred after severe bradycardia, and the R-R intervals were gradually prolonged. This case was thus apparently caused by a severe vasovagal reflex related to dysfunction in autonomic cardiac regulation and the blunting of sympathetic activation. However, because suitable mask ventilation was performed, and normal end-tidal CO_2_ and SpO_2_ levels were confirmed, the cause of cardiac arrest was determined to be factors other than hypoxia or hypercapnia.

We considered the associations of asystole and RBBB with congenital heart disease. According to a previous study, RBBB is well correlated with high pulmonary/systemic blood flow ratio in patients with ASD [13]. However, the ASD in this patient had already been surgically repaired. Another study showed that in cases with surgical VSD repair, RBBB might be associated with right ventricular dysfunction [14]. However, the authors of that study concluded that after VSD repair, biventricular systolic and diastolic dysfunction may develop irrespective of the presence of RBBB [14]. Therefore, the dysfunction may not be directly related to the RBBB. For the aforementioned reasons, we consider that RBBB with congenital heart disease may not be directly related to the asystole in the present case.

In previous studies, an anticholinergic drug was administered to counter bradycardia [5, 15]; however, it is unclear whether anticholinergic drugs have a protective effect against bradycardia in patients with Down syndrome. We postulate that it is necessary to place an catheter to quickly administer anticholinergic drugs. Alternatively, intramuscular anticholinergic drugs should be administered and the HR should increase before beginning mask induction because severe bradycardia may rapidly progress to asystole, as observed in our case.

We expected that sedation would be sufficient for intravenous and arterial line cannulation. Indeed, we considered that inducing a sufficient level of sedation would keep the patient in a calm state for any intervention. However, in this case, the administration of additional intramuscular midazolam might have resulted in a more effective outcome. Therefore, if we were reoperating on this patient under general anesthesia, we would administer sufficient quantities of intramuscular midazolam before inserting an intravenous catheter. We would subsequently increase the concentration of sevoflurane gradually or perform rapid induction with the use of intravenous anesthetics.

## Conclusions

This case demonstrates the possibility for patients with Down syndrome to experience cardiac arrest during induction of general anesthesia. We conclude that there is a possibility in pediatric cases of Down syndrome, presenting with or without heart disease, to experience transient cardiac arrest during anesthesia induction with sevoflurane.

## Abbreviations

ASD, atrial septal defect; BP, blood pressure; CPR, cardiopulmonary resuscitation; ECG, electrocardiogram; HR, heart rate; PDA, patent ductus arteriosus; RBBB, right bundle branch block; SpO_2_, peripheral oxygen saturation; VSD, ventricular septal defect
